# Optimisation of whole-body PET/CT scanning protocols

**DOI:** 10.2349/biij.3.2.e36

**Published:** 2007-04-01

**Authors:** H Zaidi

**Affiliations:** Division of Nuclear Medicine, Geneva University Hospital, Geneva, Switzerland

**Keywords:** PET/CT, data acquisition, protocol, data processing, optimisation

## Abstract

Positron emission tomography (PET) has become one of the major tools for the* in vivo* localisation of positron-emitting tracers and now is performed routinely using ^18^F-fluorodeoxyglucose (FDG) to answer important clinical questions including those in cardiology, neurology, psychiatry, and oncology. The latter application contributed largely to the wide acceptance of this imaging modality and its use in clinical diagnosis, staging, restaging, and assessment of tumour response to treatment. Dual-modality PET/CT systems have been operational for almost a decade since their inception. The complementarity between anatomic (CT) and functional or metabolic (PET) information provided in a “one-stop shop” has been the driving force of this technology. Although combined anato-metabolic imaging is an obvious choice, the way to perform imaging is still an open issue. The tracers or combinations of tracers to be used, how the imaging should be done, when contrast-enhanced CT should be performed, what are the optimal acquisition and processing protocols, are all unanswered questions. Moreover, each data acquisition–processing combination may need to be independently optimised and validated. This paper briefly reviews the basic principles of dual-modality imaging and addresses some of the practical issues involved in optimising PET/CT scanning protocols in a clinical environment.

## INTRODUCTION

Diagnosis, staging, treatment, prognosis and follow-up are the principal elements in the management of cancer, and nuclear medicine plays an important role in all these elements. Among all diagnostic and therapeutic procedures, nuclear medicine is unique in that it is based on molecular and pathophysiological mechanisms, and employs radioactively labelled biological molecules as tracers to study the pathophysiology of the tumour *in vivo* to direct treatment and assess response to therapy [1]. The specific role of PET imaging in the expansion of our understanding of the pathophysiological mechanisms of cancer and in the clinical management of patients is steadily progressing. PET, an imaging modality with sensitivity in the picomolar range, allows *in vivo* non-invasive 3D imaging of regional metabolism and many other physiological mechanisms. Since functional disturbances occur often earlier than structural once, a faster and more sensitive detection is possible.

Whereas the advent of dedicated dual-modality imaging systems designed specifically for clinical use is relatively recent, the potential advantages of combining anatomical and functional imaging has been recognised for several decades by pioneering radiological scientists and physicians [2]. Combining anatomical and functional or metabolic information into a fused image has been pursued for a long time. Early attempts were made by software fusion of PET/SPECT and x-ray CT/MR images [3]. However, these efforts often come across significant limitations, particularly in cases with non-explicit differential diagnosis or in parts of the body other than the brain. The coregistration of brain images is relatively straightforward owing to its rigid structure, whereas especially in the abdomen or thorax an exact repositioning of the patient on two different scanners (usually physically located in two different departments involving different operators) is tricky and makes the precise alignment of images from two modalities doubtful [4]. However, in any case a hardware combination in a single gantry of multimodal imaging devices ensures a much better alignment of the images and gives much higher confidence to the clinicians [5]. A hardware combination of imaging modalities (e.g. PET/CT) not only provides optimally aligned images, but also simplifies the logistics of scheduling and organising patients’ scanning given that PET/CT presents the opportunity for a ‘one stop-shopping’ approach [6].

Although combined anato-molecular imaging is an obvious choice, the design of specific clinical protocols and flexible workflow utilities is still under development and open to debate. The tracers or combinations of tracers to be used, when and how the imaging should be done, the selection of optimal acquisition, processing and display protocols, and the method of accurately performing quantitative analysis of data are still undetermined. This review documents technological advancement of the field of PET/CT imaging where special emphasis is put on optimised clinical data acquisition protocols and strategies to reduce artefacts and interpretative pitfalls.

## PRINCIPLES OF PET/CT: THEORY AND PRACTICE

The first combined PET/CT prototype allowing the acquisition of functional and anatomical images in a single session on the same scanner bed was developed in the late 1990s by investigators from the University of Pittsburgh [7]. This hybrid unit consists of two separate devices, namely a PET and a CT scanner, linked by one common bed and workstation console where data from both modalities are acquired sequentially rather than simultaneously as planned during the earlier conceptual design of the machine [8]. Both the CT components and the PET detectors were mounted on opposite sides of the rotating stage of the CT system, and imaged a patient with a common patient table translated between the centres of the two tomographs which are offset axially by 60 cm. The PET/CT system has a specially designed patient table that is designed to minimize deflection when it is extended into the patient port. The PET/CT prototype was operational at the University of Pittsburgh from May 1998 to August 2001, during which over 300 cancer patients were scanned [9]. The success of these initial studies prompted significant interest from the major medical imaging equipment manufacturers who now all have introduced commercial PET/CT scanners for clinical use.

Commercial PET/CT systems are usually configured by designing a gantry that mounts a stationary PET detector ring in tandem with a platform that rotates the CT imaging chain around the patient using a mechanical configuration similar to that used in a conventional diagnostic CT scanner. The CT study typically is used for both localisation of the FDG uptake as well as for attenuation correction of the PET data set. Besides, the use of CT in comparison to radionuclide transmission sources for producing the attenuation data increases patient throughput by approximately 30% [10]. However, CT also increases patient dose and despite the significant progress achieved in CT-based attenuation correction (CT-AC) during the last decade, some problematic issues still remain open research questions and are being investigated by many active research groups [11, 12].

The major area of clinical use of PET/CT is in oncology, where the most commonly used radiopharmaceutical is ^18^F-fluorodeoxyglucose (FDG). FDG-PET has already had a huge valuable outcome on cancer treatment and its use in clinical oncology practice continues to develop [13, 14]. The advantages of combining morphological and functional imaging (compared to PET or CT alone) have been clearly demonstrated by numerous publications for a wide variety of applications [9, 15-17]. There is an abundant literature reporting patient studies where the combined PET/CT images provided additional information, thus impacting the characterisation of abnormal FDG uptake and influencing patient management.

The recent progress in the development of tracers targeted to other aspects of tumour biology, including cell growth, cell death, oncogene expression, drug delivery, and tumour hypoxia will significantly enhance the capability of clinical scientists to differentiate tumours and are likely to be used to guide treatment decisions. The contribution of PET to understanding the clinical biology of cancer and to guiding targeted, individualised therapy will continue to grow with these new developments [18, 19]. Central to this expanding role in oncology will be the ability to make quantitative interpretations of the PET imaging data [1].

## STANDARD PET/CT SCANNING PROTOCOLS

**Figure 1:**
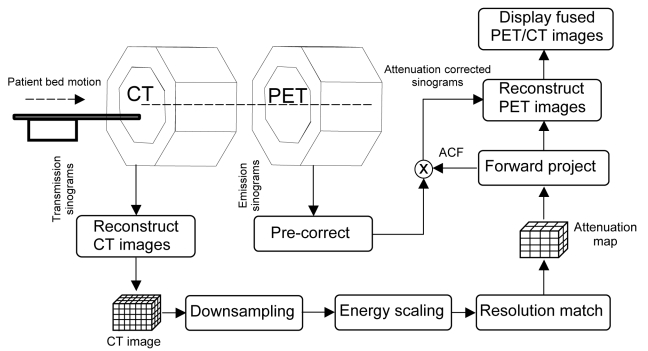
Principles of a typical PET/CT data acquisition protocol showing the main hardware components of a hybrid imaging system and the major steps involved for generating the attenuation map required for CT-based attenuation correction.

Figure 1 shows the essential steps that comprise a typical PET/CT scan, demonstrating the degree of integration available in a modern dual-modality imaging system [20]. (i) The patient is prepared for imaging which commonly includes administration both with contrast media [21] and with the radiopharmaceutical, typically 370 to 555 MBq (10 to 15 mCi) of ^18^F-FDG in adults. (ii) The patient then is asked to remove all metal objects that could introduce artefacts in the CT scan and then is positioned on the patient table of the dual/modality imaging system. (iii) The patient then undergoes an “overview” or “scout” scan during which x-ray projection data are obtained from the patient to identify the axial extent of the CT and PET study. (iv) The patient undergoes a CT acquisition. (v) The patient then undergoes the nuclear medicine study approximately 1 hour after FDG administration. (vi) The CT and PET data then are reconstructed and registered, with the CT data used for attenuation correction of the reconstructed PET tomograms. (vii) The images are reviewed by a physician who can view the CT scan, the PET images, and the fused x-ray/radionuclide data, followed by preparation of the associated clinical report.

In practice, however, running a PET/CT scanner in a clinical environment to the uppermost diagnostic standards is not straightforward. Translating the experience and know-how gained in radiology to a nuclear medicine department and vice versa is not that easy owing to the controversies surrounding PET/CT and the existing territorial and protective practices in health care facilities. Careful patient preparation and positioning are key elements of the long chain of data acquisition and processing protocols and require extensive training of technologists operating the scanner to minimize artifacts and reduce interpretative pitfalls.

**Figure 2:**
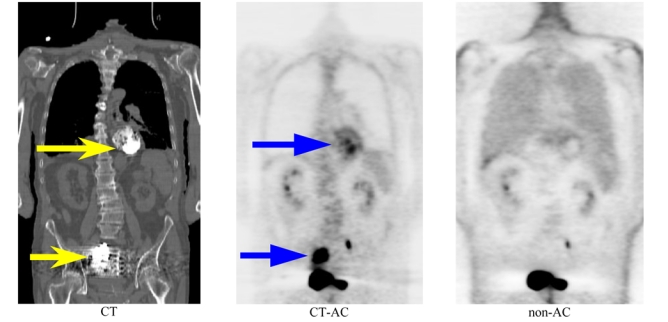
Oral contrast-enhanced related artefact in clinical PET/CT imaging. The region concentrating oral contrast shown on CT (left, arrows) led to areas of apparently increased glucose metabolism on CT-based attenuation corrected PET (centre, arrows). On fused PET/CT images, this area of apparently increased glucose metabolism correlated with high-density oral contrast on CT (not shown). Reconstructed PET images without attenuation correction demonstrated absence of lesions (right), demasking areas of apparently increased glucose metabolism as artefact. Courtesy of Prof. H. Abdel-Dayem.

As mentioned above, notwithstanding the success and widespread clinical adoption of PET/CT, there are several challenges that face the use of dual-modality imaging, and that may represent inherent limitations in this technique. In addition to a much higher absorbed dose to the patient, there are many physical and physiological factors that hamper the accurate registration of both imaging modalities and the accurate quantitative analysis of PET data following CT-AC including the inherent difference between CT and PET image matrix size and resolution, polychromaticity of x-ray photons (30-140 keV) requiring transformation to monoenergetic 511 keV photons [22], misregistration between CT and PET images resulting for instance from respiratory motion [23-26], truncation artefacts owing to discrepancy between fields of view in a combined PET/CT scanner [27-29], the presence of oral and intravenous contrast medium [21, 30-38], artefacts due to metallic implants [39-46], beam hardening [47, 48], x-ray scatter in CT images for future generation cone-beam geometries [49-51], and other CT artefacts from any source. As an example, figure 2 illustrates typical artefacts resulting from the presence of oral contrast medium during PET scanning when using CT-based attenuation correction in PET.

In particular, metal artefacts are a major problem in CT. They are due to the presence of strongly attenuating objects in the field-of-view. The presence of metallic dental implants can also introduce artefacts into brain images, not only when CT is used to determine the attenuation map in PET/CT, but also when a standard positron source is employed for attenuation correction [44]. A limited number of studies reported in the literature detailed comparative assessment studies between CT-AC and radionuclide scanning-based AC including ^68^Ga vs CT-AC and ^137^Cs vs. CT-AC [12]. The most important causes of metal artefacts are: noise, beam hardening, the non-linear partial volume effect, and scatter. In order to develop new algorithms for reduction of metal artefacts, one usually hypothesize that artefacts are due to deviations of the acquisition model assumed by the reconstruction from the true acquisition process. Consequently, improving the acquisition model should reduce artefacts.

Qualitative visual assessment remains the principal method followed in the interpretation of routine clinical PET studies. Qualitative interpretation of clinical FDG-PET scans is usually based on the identification of regional glycolysis through a differential assessment of the contrast between sites of tracer uptake resulting from a normal physiological process or a pathological state compared to the surrounding background. However, visual interpretation intrinsically bears many important weaknesses including the need to define a threshold for judgment of the existence and degree of radiotracer concentration among other physical and physiological factors, issues related to inter- and intra-observer reliability for qualitative assessment in clinical trials, …etc. Therefore, despite its simplicity, critical role and wide adoption in the daily clinical practice, visual interpretation has many fundamental shortcomings which limit its role in research studies where more emphasis is put on quantitative measures that allow more objective and reliable assessment.

Currently, the standardised uptake value (SUV) continues to be the most widely used uptake index in clinical PET studies. This semi-quantitative parameter is defined as the tissue concentration of tracer within a lesion divided by tissue density, as measured by PET, divided by the injected dose normalised to patient weight multiplied by a decay factor [52]. In practice, the SUV is calculated by dividing the activity concentration in the region of interest (ROI) drawn around the lesion (MBq/mL) by the injected dose (MBq) divided by the body weight (g):

$$SUV\;=\;\frac{\frac{Mean\;activity\;concentration\;(MBq/cc)}{Tissue\;density\;(g/cc)}}{\frac{Injected\;dose\;(MBq)}{Body\;weight(g)}}\times \frac{1}{decay\;factor}$$

Since the weight is not always a good measure of initial tracer distribution volume, several investigators suggested variants on the SUV to account for this effect particularly for obese patients. This includes SUV using lean-body mass (lean) [53] or body surface area (BSA) [54] in place of patient weight in the equation above, yielding SUV_lean_ and SUV_BSA_, respectively, to reduce the variation of SUV associated to patient’s body composition and habitus. For research studies, simplified and more rigorous tracer kinetic analysis techniques are usually adopted [55].

**Figure 3:**
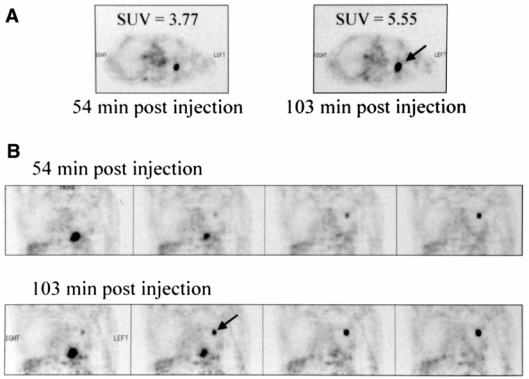
Comparison of early and delayed FDG-PET images from a lung cancer patient. transaxial images (A) and coronal images (B). Arrow points to lesion. Malignant focus became more apparent in later images and SUV increased from 3.77 to 5.55. Reprinted with permission from [56].

In addition to the factors discussed above, it has been reported in many studies that variations in the time interval between tracer injection and PET scanning (uptake period) considerably influence SUV estimation [55, 56]. It should be emphasised that in many of these studies, dual-time point PET improved both the sensitivity and the specificity of PET for a variety of malignancies, including breast cancer [57-59], lung nodules [60], head and neck cancer [61] and gallbladder carcinoma [62]. In theory, this is the result of two factors: firstly the sustained augmented FDG uptake in malignant lesions allows to discriminate them with higher specificity, and secondly, enhanced lesion-to-background contrast leads to improved lesion detectability (Fig. 3). The later is the result of a combination of FDG washout from neighbouring normal tissues and enhanced FDG uptake in the lesion. This is remarkable given that there is always a trade-off between sensitivity and specificity for the majority of other diagnostic imaging investigations, frequently suggesting that improvement in performance of one parameter can be achieved only at the detriment of the second and vice versa [55].

## OPTIMISATION OF PET/CT SCANNING PROTOCOLS

**Figure 4:**
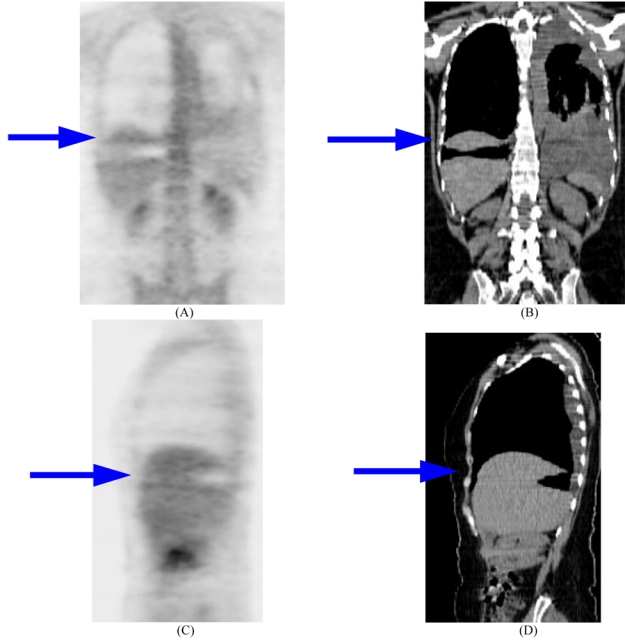
Illustration of a respiratory motion related artefact on PET images reconstructed with CT-based attenuation correction. (A) coronal ^18^F-FDG PET, (B) Coronal CT, and (C) sagittal ^18^F-FDG PET, and (D) sagittal CT. A region of decreased metabolic activity is demonstrated in the diaphragmatic region (horizontal arrow), representing a “cold artefact”.

**Figure 5:**
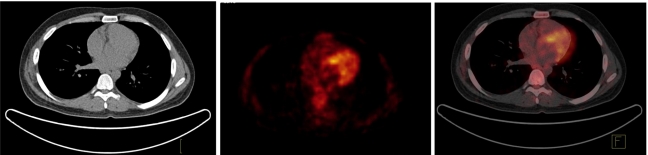
Illustration of a cardiac motion related artefact on PET images reconstructed with CT-based attenuation correction showing the anatomical CT images (left), PET image (centre), and the fused PET/CT image (right).

Despite the fact that PET/CT became the de facto standard for clinical PET imaging, there are several challenges that face its use and that may represent inherent limitations in this technique. All commercially available PET/CT systems record the emission and transmission data using different detectors instead of a single detector. Moreover, the x-ray and PET imaging chains are separated by a non-negligible distance, to facilitate mechanical clearance and to avoid blinding and damaging the PET detectors and contaminating the x-ray CT data by scatter radiation emanating from the emission PET scan. One probable trouble arises when the patient moves either voluntarily or involuntarily between or during the CT and PET data acquisitions. This might take place, for instance, if the patient changes his position while lying on the patient bed. Patient motion might also occur due to respiration, cardiac motion, peristalsis, and bladder filling, all of which can lead to motion blurring or misregistration errors between PET and CT data [17]. Diagnostic quality CT data are usually acquired using a breath-hold protocol, whereas PET data are acquired over several minutes with the patient breathing softly. Differences between PET and CT breathing protocols might lead to misalignment artefacts owing to anatomical dislocations of the diaphragm and chest wall during a PET/CT scan. A slight displacement of the diaphragm’s position on the CT scan can cause a substantial bias in the estimation of the tracer concentration in the reconstructed PET data when the former is used for attenuation correction [63]. The outcome of an inconsistency in diaphragmatic location between PET and CT is frequently the appearance of the so-called “cold” artefact at the lung base (Fig. 4). Many studies reported significant misalignment between the CT and the PET data. For example, in a study of 300 clinical PET/CT studies with proven liver lesions; approximately 2% appeared to have the lesion localised in the lung [64] whereas the misalignment between PET and CT data was greater than 2 cm in 34 of 100 patient studies due to respiratory motion [65]. Cardiac motion can also be a source of misregistration between the CT and PET images (Fig. 5).

Caution is therefore commended when reading PET/CT scans of patients suffering from disease in periphery of the lung where noticeable tracer uptake can be the result of respiratory motion rather than disease. Modern PET/CT scanners are equipped with helical CT technology allowing to acquire high resolution anatomical images within a few seconds following patient positioning and definition of the axial field of view on the topogram. It is therefore obvious that PET is the limiting factor when it comes to scanning speed on combined PET/CT. Whenever faster scanning times are sought, PET is the imaging modality requiring improvement through the development of novel detector technologies, faster scintillation crystals and electronic boards, new geometries offering higher sensitivity and many other means that are being explored. One possibility would be to substitute conventional PET detector blocks with LSO panel detectors [66] covering a larger axial field of view with the aim of achieving faster scan times than are achievable with current systems. In any case, faster scan times improve both patient comfort and reduce the time during which patient motion can occur. Likewise, faster scan times can increase patient throughput and thereby boost system utilisation and improve cost-effectiveness.

The progress in CT-AC methodology has been immense in the last few years, the main opportunities arising from the development of both optimised scanning protocols and innovative and faster image processing algorithms. This has permitted the implementation of much more ambitious algorithms that tackle the challenges of whole-body imaging using PET. Some solutions were recently proposed and used successfully in clinical and research settings. This includes optimised contrast-enhanced CT protocols [38, 67], respiratory motion [65, 68, 69], metal artefacts reduction [70-89], truncation artefacts correction [27-29], beam hardening [47, 48] and x-ray scatter [49-51]. These hot topics undoubtedly still require further research and development efforts.

## CHALLENGES AND FUTURE DIRECTIONS

One decade elapsed since the introduction of dual-modality PET/CT imaging in clinical routine. The supporters of this imaging modality claim that the barriers for wider adoption of this technology were driven by bureaucratic and protective motivations rather by scientific reasons [90]. Still there are many technical issues that need to be solved through research [91]. Despite much worthwhile research performed during the last few years, artefacts induced by respiratory motion remain among the most difficult problems to solve [92, 93]. Another limitation of current PET/CT technology is that sequential rather than simultaneous data acquisition is performed [11].

Sequential scanning renders an accurate temporal correlation of non-repeatable functional *in vivo* processes impractical, which is a major restriction of current generation PET/CT scanners [1]. Moreover, CT has low soft tissue contrast and delivers pretty high absorbed radiation doses, which can result in noticeable biological effects, a rather serious issue particularly in paediatric studies. This might also change the animal model being studied in preclinical research using molecular imaging techniques ending up with unreliable results. More importantly, owing to its low sensitivity, perfusion is the only *in vivo* functional information provided by CT in contrast enhanced studies. This is in contrast to capabilities and the wealth of information offered by MRI (in addition to higher soft tissue contrast) through fMRI and MR spectroscopy to enhance the diagnostic performance and quantitative capabilities of PET [3, 94]. Whether PET/MR will succeed to replace PET/CT as the multimodality molecular imaging platform of choice in the future is still an open and important question that will retain the attention of active researchers in the field during the next decade [95, 96].
